# A one-pot cascade protocol for diarylation of amines and water

**DOI:** 10.1016/j.xpro.2022.101700

**Published:** 2022-09-22

**Authors:** Erika Linde, Berit Olofsson

**Affiliations:** 1 Department of Organic Chemistry, Arrhenius Laboratory, Stockholm University, 106 91 Stockholm, Sweden

**Keywords:** Chemistry, Material sciences

## Abstract

N- and O-arylated compounds are prevalent in pharmaceuticals and materials, and efficient approaches for their synthesis are important. Herein, we present an efficient protocol for the diarylation of aliphatic amines and water with two structurally different aryl groups in one single step, yielding highly functionalized diaryl amines and ethers. We describe the synthesis of the required diaryliodonium salts and detail the procedure for the diarylation. The protocol is limited to use of unhindered amines and diaryliodonium salts with certain substituents.

For complete details on the use and execution of this protocol, please refer to Linde et al. (2022).

## Highlights

• SNAr reactivity with *ortho*-fluorinated diaryliodonium salts
• Atom-efficient diarylation of primary amines, ammonia, and water
• Convenient and simple route to diarylamines and diaryl ethers
• Facile synthesis of the diaryliodonium salts from iodoarenes and arenes

## Abstract (editor highlights)

Publisher’s note: Undertaking any experimental protocol requires adherence to local institutional guidelines for laboratory safety and ethics.

## Graphical abstract

**Figure:**
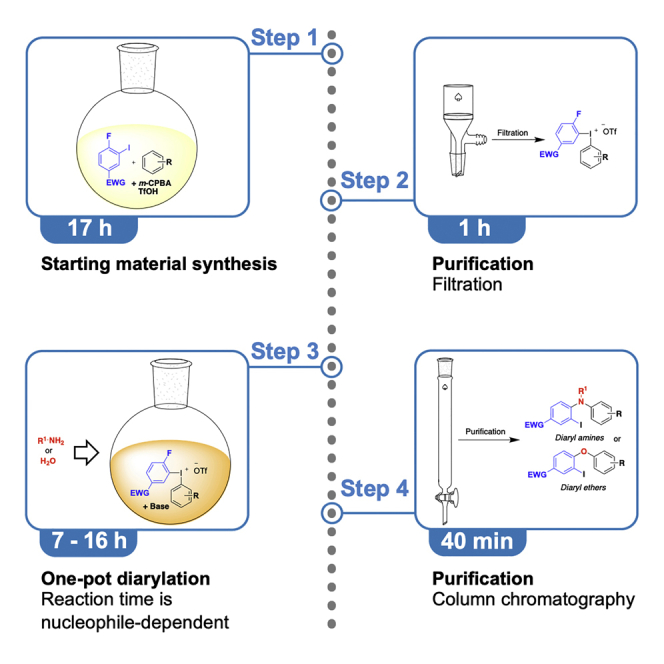

## Before you begin

Diarylamines and diaryl ethers are valuable structural motifs in pharmaceuticals, materials and agrochemicals (Chen et al., 2020; Ruiz-Castillo and Buchwald, 2016; Wang et al., 2016). The preparation of these compound classes through transition metal-catalyzed arylations (Forero-Cortés and Haydl, 2019; Sambiagio et al., 2014; West et al., 2019) is efficient and well established, but also associated with high cost, excess reagents and sometimes need for designer ligands. Nucleophilic aromatic substitution reactions (S_N_Ar) are transition metal-free, efficient and highly atom economical, but generally limited to substrates bearing strong electron withdrawing groups (EWG), or require strong bases under harsh reaction conditions (Brown and Boström, 2016). Diaryliodonium salts (Ar_2_IX) are bench-top stable and non-hazardous electrophilic arylating reagents that can be utilized under mild and transition metal-free conditions (Villo and Olofsson, 2019; Yoshimura and Zhdankin, 2016). However, stochiometric ArI waste is generally generated in the reactions (Boelke et al., 2018; Wang et al., 2018).

**Scheme 1:**
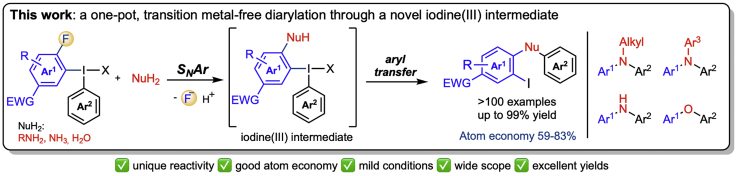
Diarylation of N- and O- nucleophiles Adapted with permission from Linde et al. (2022).

This protocol combines the atom efficiency of S_N_Ar arylations with the broad scope and mild conditions of Ar_2_IX arylations by using certain fluorinated diaryliodonium salts to synthesize diarylamines and diaryl ethers in one step. The diaryliodonium salt has one aryl group bearing an EWG as well as a leaving group that enables an initial S_N_AR arylation of the nucleophile followed by an intramolecular aryl transfer to give the diarylated nucleophile (Scheme 1). The preparation of the diaryliodonium salts is described within this protocol as well as the diarylation of amines and water. We have used similar conditions to prepare triarylamines from anilines, and diarylamines from ammonia, as depicted in Scheme 1 and described in Linde et al. (2022).

1. Dry the m-chloroperbenzoic acid (*m*-CPBA) used for the synthesis of the diaryliodonium salts under vacuum for 1 h at the Schlenk-line, and titrate it as described in (A. I. Vogel et al., 1996).
2. Distill the amine, unless you take it from a new bottle.
3. Degass the deionized water prior to use by argon bubbling.
  a. Keep the water in a pear-shaped flask sealed by a septum, and put a long needle connected to a Schlenk-line through the septum into the solvent.
  b. Add a needle outlet on the septum to allow the oxygen to escape from the flask when argon is bubbled through the solvent. Store the water under argon atmosphere for up to a week.
4. Use flame- or oven dried reaction vessels for the diarylation reactions. Use microwave vials for small-scale reactions (0.1–1.0 mmol) and round bottom flasks for larger scale reactions.
5. Use anhydrous solvents obtained from VAC solvent purification systems for the diarylation reactions. Degass the solvents prior to every reaction by argon bubbling for 15 min.

## Key resources table

**Table:** 

REAGENT or RESOURCE	SOURCE	IDENTIFIER
**Chemicals, peptides, and recombinant proteins**		

4-Fluoro-3-iodonitrobenzene	Fluorochem	CAS 177363-10-9
Benzene	Sigma-Aldrich	CAS 71-43-2
*m*-CPBA	Sigma-Aldrich	CAS 937-14-4
Triflic acid	Fluorochem	CAS 1493-13-6
3-phenylpropylamine	Sigma-Aldrich	CAS 2038-57-5
Water	Deionized water tap in the lab	n/a
Potassium carbonate	Sigma-Aldrich	CAS 584-08-7
Cesium carbonate	Sigma-Aldrich	CAS 534-17-8
Dichloromethane	Honeywell Riedel-de-Haën	CAS 75-09-2
Acetonitrile	Honeywell Riedel-de-Haën	CAS 75-05-6
Ethyl acetate	Sigma-Aldrich	CAS 141-78-6
Diethyl ether	Honeywell Riedel-de-Haën	CAS 60-29-7
Pentane	VWR	CAS 109-66-0
Silica gel 40–63 μm	VWR	CAS 7631-86-9

**Other**		

Round bottom flask (250 mL)	Rettberg	Cat# 134020236
Septa (30 mm)	VWR	Cat# 217-0186
Microwave vials (5 mL)	Rettberg	Cat# 12992069
Caps for microwave vials	Scantec Nordic	Cat# 69.20030142
Hamilton syringes 10 μL	Rettberg	Cat# 81008
Hamilton syringes 1,000 μL	Rettberg	Cat# 81301
Vacuum/Argon- Schlenk manifold	Rettberg	Cat# 134030100
Stirring/heating plate	IKA	Cat# 0003810000
Glass filter	Rettberg	Cat# 258522102
Glass column	Rettberg	Cat# 867000311

## Step-by-step method details

### Synthesis of diaryliodonium salts

**Timing: 18 h**

**Scheme 2:**
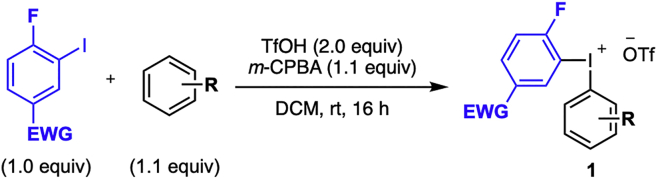
General synthesis of diaryliodonium salts

In this step, the diaryliodonium salts are synthesized by a one-pot method following a literature procedure developed by our group (Bielawski et al., 2007). This procedure is depicted in Scheme 2 and can be used without modifications for other electron-deficient fluorinated iodoarenes and the full scope of diaryliodonium salts is available at Linde et al. (2022).

**Scheme 3:**
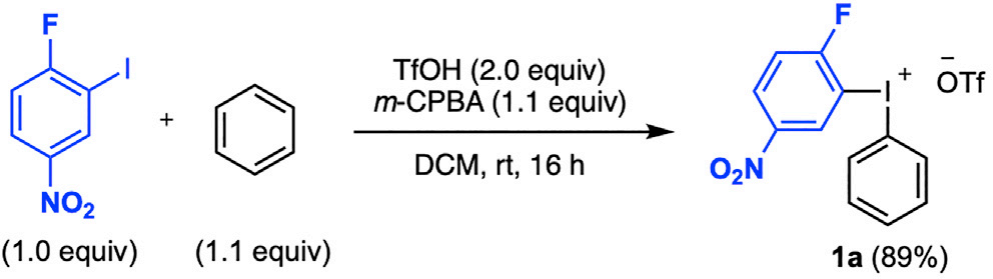
Synthesis of diaryliodonium salt **1a**

1. Reaction set up (Table 1, Scheme 3).
  a. Weigh out 4-fluoro-3-iodonitrobenzene (5.6 g, 21.0 mmol) and *m*-CPBA (5.0 g, 23.0 mmol) and add to a 250 mL round bottom flask.
  b. Add CH_2_Cl_2_ [105 mL, 0.2 M] and place the flask in an ice/ water bath at 0°C.
  c. Add trifluoromethanesulfonic acid (TfOH, 3.7 mL, 42.0 mmol) drop wise with a 10 mL Hamilton syringe, which causes the reaction to shift from colorless to yellow.
  d. Stir the reaction at 0°C until it has stopped fuming (5–10 min).
    **CRITICAL:** The quality of the *m*-CPBA is important for the reproducibility of the reaction. Always dry and titrate this reagent prior to use.
  e. Add benzene (2.1 mL, 23.0 mmol) drop wise with a syringe.
  f. Allow the reaction to reach 20°C–25°C and let it stir for 16 h.
    ***Note:*** After a few hours, a white precipitate becomes visible in the flask.
    ***Note:*** This procedure can be used without modifications for other arenes than benzene.
2. Purification.
  a. Remove the solvent by rotary evaporation to dryness.
  b. Add diethyl ether (5 mL/mmol ArI) to the crude mixture to initiate precipitation of the product.
  c. Stir the mixture at 20°C–25°C for 10 min.
  d. Close the flask with a glass or rubber stopper and store it in the freezer for 1 h.
    ***Note:*** If the precipitation of the product is difficult, see the troubleshooting 1.
  e. Take the flask out of the freezer and directly collect the product by filtration on a glass filter funnel porosity 3.
  f. Wash the product several times with diethyl ether (2–5 mL/mmol ArI).
  g. Dry the product under water-suction induced vacuum that is connected to the glass filter funnel.
  h. Transfer and weigh the product into a vial in order to calculate the yield.
  i. Analyze the product by ^1^H-, ^13^C-, ^19^F- NMR, melting point and HRMS.

**Table 1:** Quantification of reagents, solvent, and product

Reagent	Mw (g/mol)	m (g)	n (mmol)	Equiv.	V (mL)	Conc (M)	Density (g/mL)	Yield (%)
4-Fluoro-3-iodonitrobenzene	267	5.6	21.0	1.0				
Benzene	78		23.0	1.1	2.1		0.87	
*m*-CPBA (80%)	173	5.0	23.0	1.1				
TfOH	150		42.0	2.0	3.7		1.67	
DCM					105	0.2		
**1a**^a^	493	9.2	18.7					89

a The product is benchtop stable. Can be stored at 20°C–25°C without any precautions to avoid moist and/or air.

#### Analytical data

M.p.: 136.8°C; ^1^H NMR (400 MHz, DMSO-*d*_*6*_) δ 9.41 (dd, *J* = 4.9, 2.8 Hz, 1H), 8.56 (ddd, *J* = 9.1, 4.9, 2.8 Hz, 1H), 8.32 (d, *J* = 8.1 Hz, 2H), 7.85 (dd, *J* = 9.1, 7.5 Hz, 1H), 7.70 (t, *J* = 7.9 Hz, 1H), 7.57 (t, *J* = 7.8 Hz, 2H); ^13^C NMR (101 MHz, DMSO-*d*_6_) δ 162.8 (d, *J* = 258.1 Hz), 144.9, 135.3, 132.7 (d, *J* = 3.1 Hz), 132.1, 132.1, 130.9 (d, *J* = 10.5 Hz), 120.7 (q, *J* = 322.4 Hz, CF_3_SO_3_^-^), 117.9 (d, *J* = 25.7 Hz), 117.2, 104.6 (d, *J* = 27.1 Hz); ^19^F NMR (377 MHz, DMSO-*d*_*6*_) δ -77.75 (s, 3F), -88.79 (s, 1F); HRMS (ESI): calc’d for C_12_H_8_FINO_2_ [M-OTf]^+^: 343.9578; found: 343.9578.

### Diarylation of aliphatic amines

**Timing: 8 h**

**Scheme 4:**
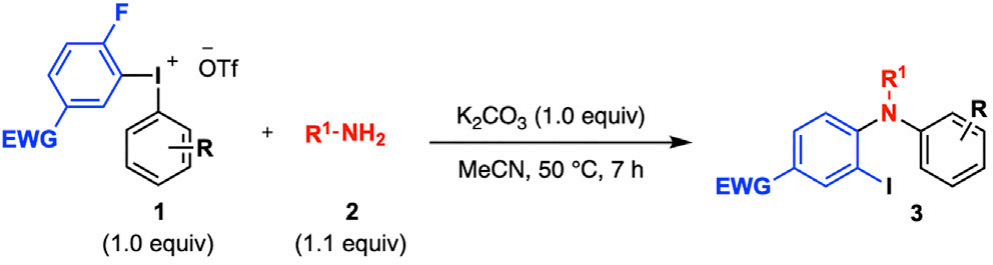
General diarylation of aliphatic amines

In this reaction, diarylamines are prepared by a one-pot procedure from diaryliodonium salts of the general structure **1** following Linde et al. (2022) (Scheme 4). Reagent **1** is reacted with the aliphatic amine and the base K_2_CO_3_ in a microwave vial under argon atmosphere. Upon completion of the reaction, the product is isolated through column chromatography without prior work up. The full scope of the reaction is described in Linde et al. (2022).

**Figure 1:**
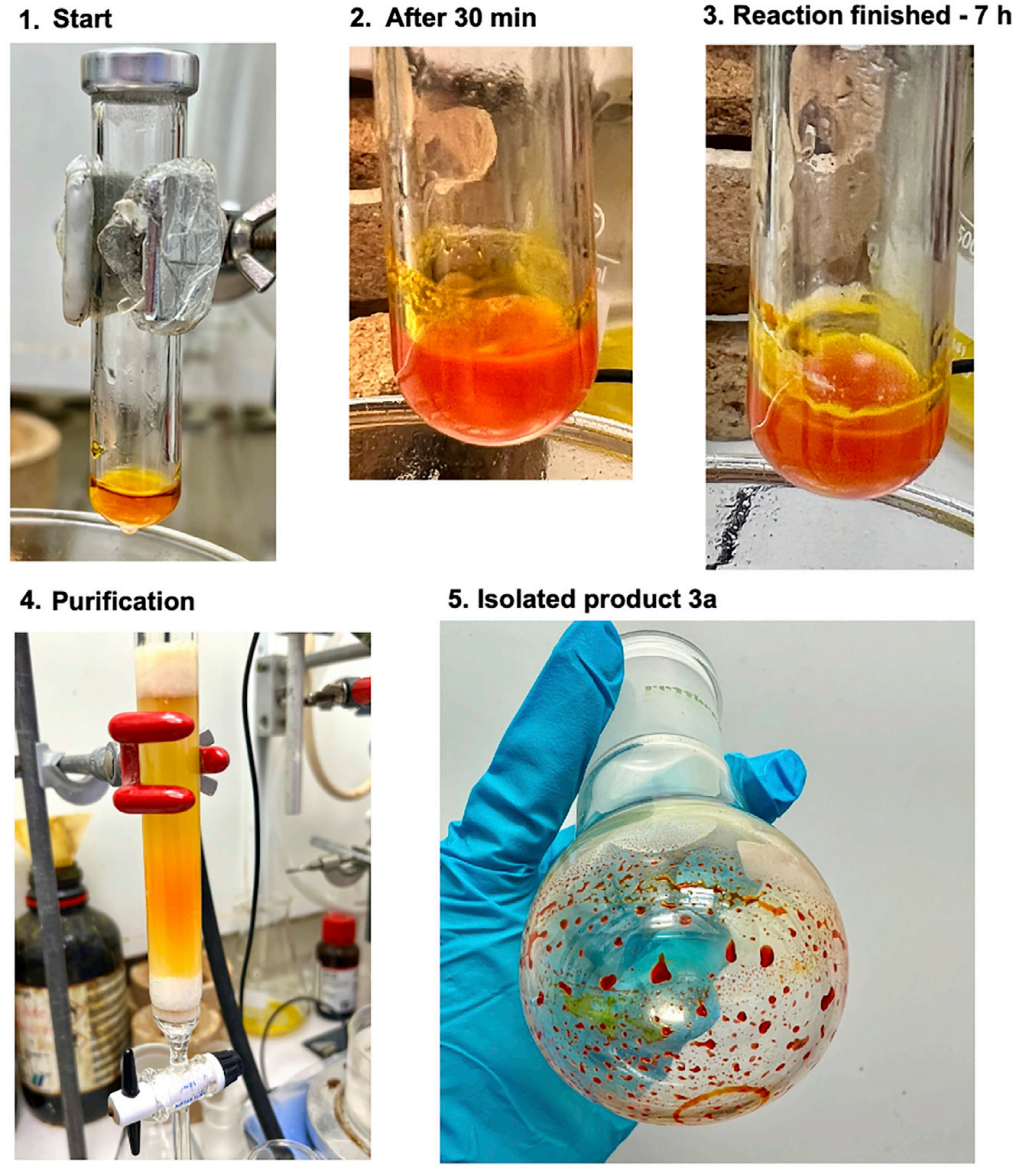
Reaction and appearance of product **3a** at different stages of the reaction
  g. Stir the reaction at this temperature for 7 h.

3. **Reaction set up** (Table 2, Scheme 5).
  a. Add the diaryliodonium salt (49 mg, 0.1 mmol) and the K_2_CO_3_ (14 mg, 0.1 mmol) to a flame-dried 10 mL microwave vial, and cap it.
  b. Connect the vial to a Schlenk-line and place it under vacuum.
  c. Back-flush the vial with argon and repeat this vacuum-argon cycle 3 times.
  d. Add anhydrous and degassed MeCN (0.5 mL, 0.2 M) to the vial with a plastic syringe.
  e. Add freshly distilled amine (15 μL, 0.11 mmol, 1.1 equiv) with a micro syringe.
  f. Transfer the vial to a preheated oil bath set to 50°C, which results in a color change from light yellow to red (see Figure 1).

**Table 2:** Quantification of reagents, solvent, and product

Reagent	Mw (g/mol)	m (mg)	n (mmol)	Equiv.	V (mL)	Conc (M)	Density (g/mL)	Yield (%)
**1a**	493	49	0.1	1.0				
**2a**	135		0.11	1.1	0.015		0.951	
K_2_CO_3_	138	14	0.1	1.0				
MeCN					0.5	0.2		
**3a**	458	43	0.093					93

^a^The product is benchtop stable. Can be stored at 20°C–25°C without any precautions to avoid moist and/or air.

**Scheme 5:**
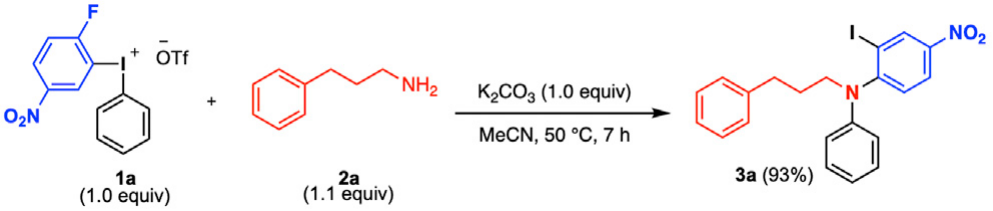
Diarylation of aliphatic amine **2a** to yield diarylamine **3a**
4. Purification.
  a. Remove the reaction vial from the warm oil bath and allow it to reach 20°C–25°C.
    Analyze the reaction by TLC with 2% diethyl ether in pentane as eluent. The product has an R_*f*_ value of 0.33.
    ***Note:*** The TLC displays three spots; the diaryliodonium salt appears at the base-line of the TLC, the product at R_*f*_ = 0.33 and traces of the reduced ArI at the top of the TLC plate, R_*f*_ = 0.9–1.
  b. Load the reaction mixture with a pipette to a silica gel column (Ø = 10 mm, height of silica added: 8.5 cm).
  c. Use 1 mL of the eluent (2% diethyl ether in pentane) to rinse the microwave vial and then add the solution to the silica gel column.
  d. Repeat the rinsing of the vial 3 times.
  e. Elute the product off the column with 100–150 mL eluent by applying a light airflow and collect the fractions in test tubes. The product appears as a red band on the column, see Figure 1 stage 4.
    ***Note:*** The flash chromatography separation is easily achieved since the major side-product is generally traces of the reduced ArI (4-fluoro-3-iodonitrobenzene), which elutes before the product off the column.
  f. Use TLC (2% diethyl ether in pentane) with UV-light detection to analyze the fractions.
  g. Collect the fractions containing the product, (R_*f*_ = 0.33).
  h. Remove the solvent under rotary evaporation to give the pure product.
    ***Note:*** If the yield of the reaction is lower than expected, see troubleshooting 2.
  i. Identify and characterize the product by ^1^H-, ^13^C- NMR, melting point and HRMS.

#### Analytical data

The product 2-iodo-4-nitro-*N*- phenyl-*N*-(3-phenylpropyl)aniline **(3a)** is obtained as an orange oil that becomes an orange solid upon storage in the fridge. The product in obtained in 93% yield at 0.1 mmol scale; M.p.: 91.3°C; R_*f*_ = 0.33 (2% diethyl ether in pentane) ^1^H NMR (400 MHz, CDCl_3_) δ 8.78 (d, *J* = 2.6 Hz, 1H), 8.24 (dd, *J* = 8.8, 2.6 Hz, 1H), 7.39–7.14 (m, 8H), 6.92 (t, *J* = 7.3 Hz, 1H), 6.64 (d, *J* = 8.7 Hz, 2H), 3.81–3.69 (m, 2H), 2.72 (t, *J* = 7.6 Hz, 2H), 2.05 (p, *J* = 7.7 Hz, 2H); ^13^C NMR (101 MHz, CDCl_3_) δ 155.5, 147.0, 144.4, 141.1, 136.6, 129.4, 128.6, 128.5, 128.1, 126.3, 124.9, 120.7, 117.7, 98.5, 52.5, 33.2, 29.3; HRMS (ESI): calc’d for C_21_H_19_IN_2_O_2_Na [M + Na]^+^: 481.0383; found: 481.0389.

### Diarylation of water

**Timing: 17 h**

**Scheme 6:**
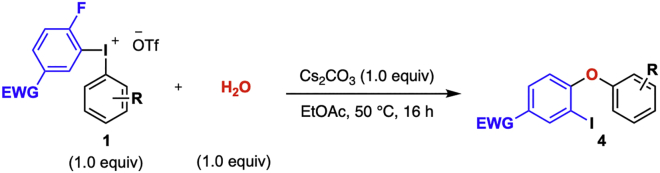
General diarylation of water

In this reaction, diaryl ethers are prepared by a one-pot procedure from diaryliodonium salts of general structure **1** following Linde et al. (2022) (Scheme 6). Reagent **1** is reacted with water and Cs_2_CO_3_ in a microwave vial under argon atmosphere. Upon completion of the reaction, the product is isolated through column chromatography without prior work up. The full scope of the reaction is described in Linde et al. (2022).

**Scheme 7:**
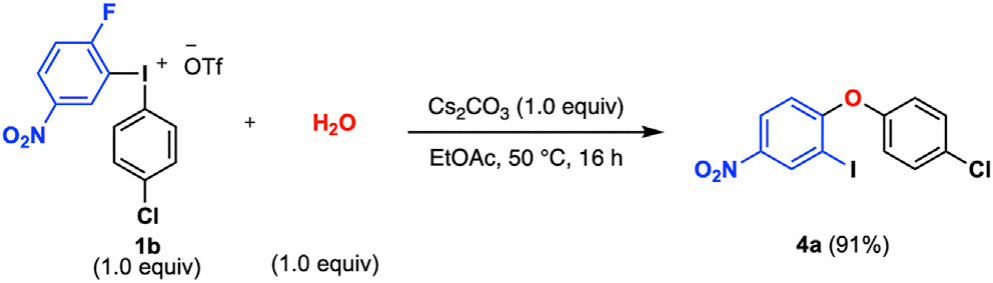
Diarylation of water to yield diaryl ether **4a**

5. Reaction set-up (Table 3, Scheme 7).
  a. Add the diaryliodonium salt (105 mg, 0.2 mmol) and the Cs_2_CO_3_ (65 mg, 0.2 mmol) to a flame-dried 10 mL microwave vial and cap it.
  b. Connect the vial to a Schlenk-line and put under vacuum.
  c. Back-flush the vial with argon and repeat this vacuum-argon cycle 3 times.
  d. Add degassed EtOAc (1.0 mL, 0.2 M) to the vial with a plastic syringe.
  e. Add degassed deionized water (4 μL, 0.2 mmol) with a micro syringe.
    **CRITICAL:** It is crucial for the success of the reaction to avoid use of excess water since this results in a significant decrease in product yield. The quality of the Cs_2_CO_3_ is also important and should be stored in a desiccator between reactions.
  f. Transfer the vial to a preheated oil bath set to 50°C.
    ***Note:*** Diaryliodonium salts bearing electron rich aryls generally need 70°C reaction temperature.
  g. Stir the reaction at the chosen temperature for 16 h.
    ***Note:*** If the reaction does not go to completion, see troubleshooting 3.
6. Purification.
  a. Remove the reaction vial from the warm oil bath and allow it to reach 20°C–25°C.
  b. Analyze the reaction by TLC with 3% diethyl ether in pentane as eluent. The product has an R_*f*_ value of 0.15.
    ***Note:*** The TLC displays three spots; the diaryliodonium salt appears at the base-line of the TLC, the product at R_*f*_ = 0.15 and traces of the reduced ArI at the top of the TLC plate, R_*f*_ = 0.9–1.
  c. Load the reaction mixture directly with a pipette to a silica gel column (Ø = 10 mm, height of silica added: 8.5 cm).
  d. Add 1 mL of the eluent (3% diethyl ether in pentane) to the microwave vial to rinse it to make sure no product is left behind in the reaction vial.
  e. Repeat the rinsing of the vial three times.
  f. Elute the product of the column by applying a light airflow and collect the fractions in test tubes.
    ***Note:*** The separation is easily achieved since the major side-product is generally traces of the reduced ArI (4-fluoro-3-iodonitrobenzene), which elutes before the product off the column.
  g. Use TLC (3% diethyl ether in pentane) with UV-light detection to analyze the fractions.
  h. Collect the fractions containing product (R_*f*_ = 0.15).
  i. Remove the solvent under rotary evaporation to give the pure product.
    ***Note:*** If the yield of the water diarylation is lower than expected, see troubleshooting 4.
  j. Identify and characterize the product by ^1^H-, ^13^C- NMR and compare to reference data (Panda et al., 2015).

**Table 3:** Quantification of reagents, solvent, and product

Reagent	Mw (g/mol)	m (mg)	n (mmol)	Equiv.	V (mL)	Conc (M)	Density (g/mL)	Yield (%)
**1b**	527	105	0.2	1.0				
H_2_O	135		0.2	1.1	0.004		0.99	
Cs_2_CO_3_	138	65	0.2	1.0				
EtOAc					1.0	0.2		
**4a**	375	68	0.181					91

^a^The product is benchtop stable. Can be sto red at 20°C–25°C without any precautions to avoid moist and/or air.

#### Analytical data

The product 2-Iodo-4-nitrophenyl(4-chlorophenyl) ether **(4a)** is obtained as a colorless oil at 0.2 mmol scale. The product was isolated in 91% yield. On larger scale the product is obtained as a white solid; R_*f*_ = 0.15 (3% diethyl ether in pentane); ^1^H NMR (400 MHz, CDCl_3_) δ 8.74 (d, *J* = 2.7 Hz, 1H), 8.14 (dd, *J* = 9.1, 2.7 Hz, 1H), 7.41 (d, *J* = 9.0 Hz, 2H), 7.03 (d, *J* = 8.9 Hz, 2H), 6.76 (d, *J* = 9.1 Hz, 1H); ^13^C NMR (101 MHz, CDCl_3_) δ 162.3, 153.4, 143.5, 135.7, 131.1, 130.6, 125.5, 121.7, 115.7, 86.4.

## Expected outcomes

This protocol allows for efficient preparation of diarylamines and diaryl ethers in a one-pot reaction under mild and transition-metal free conditions by use of fluorinated diaryliodonium salts. The reaction set up and purification is simple and does not require excess or toxic reagents or any advanced equipment. The reaction is high yielding and displays a broad functional group tolerance which results in large synthetic flexibility. This diarylation strategy demonstrates impressive atom economy compared to conventional monoarylations with diaryliodonium salts since the iodoarene waste formation is circumvented. The diaryliodonium salts are easily prepared in high yields by a one-pot method that was previously developed in our laboratory (Bielawski et al., 2007).

## Limitations

The protocol is generally limited to the use of diaryliodonium salts bearing an EWG in either *para*- or *ortho-*position to the fluorine leaving group. Moving the EWG to a *meta*-position to the leaving group will inhibit the desired reaction pathway. The amine diarylation is generally more efficient with non-hindered aliphatic amines. Reactions with amines with a secondary α-carbon are sluggish whereas amines with tertiary α-carbons are inert. The diarylation of water has limitations concerning the diaryliodonium salt structure, due to the lower nucleophilicity of water. A relatively strong EWG is required on the fluorinated aryl group of the diaryliodonium salt. Salts bearing weaker EWGs than a CN group (Hammett constant σ_para_: 0.66) were unproductive with water as the nucleophiles, e.g., -CO_2_Me (σ_para_: 0.45) and -CF_3_ (σ_para_: 0.54) (Hansch et al., 1991). Scaling up the water arylation led in some instances to a decreased yield; as exemplified by the reaction of salt **1b** that resulted in 75% yield at 1.0 mmol scale vs 91% yield at 0.2 mmol scale.

## Troubleshooting

### Problem 1

Step 2d: The diaryliodonium salt does not precipitate (completely) upon addition of ether.

### Potential solution

To achieve full precipitation of the product, the time in the freezer can be increased to 16–24 h. If the precipitation is difficult upon addition of ether, more vigorous stirring in an ice bath can facilitate the process. If the compound is a sticky oil, it can sometimes help to add a few drops of CH_2_Cl_2_ or methanol. If the product solid is a very fine powder, decanting of the solvent can be a better option than filtration.

### Problem 2

Step 4i: The yield of a diarylation of an amine is lower than expected.

### Potential solution

Distill the amine to assure its quality. This can have a drastic effect on the yield.

### Problem 3

**Figure 2:**
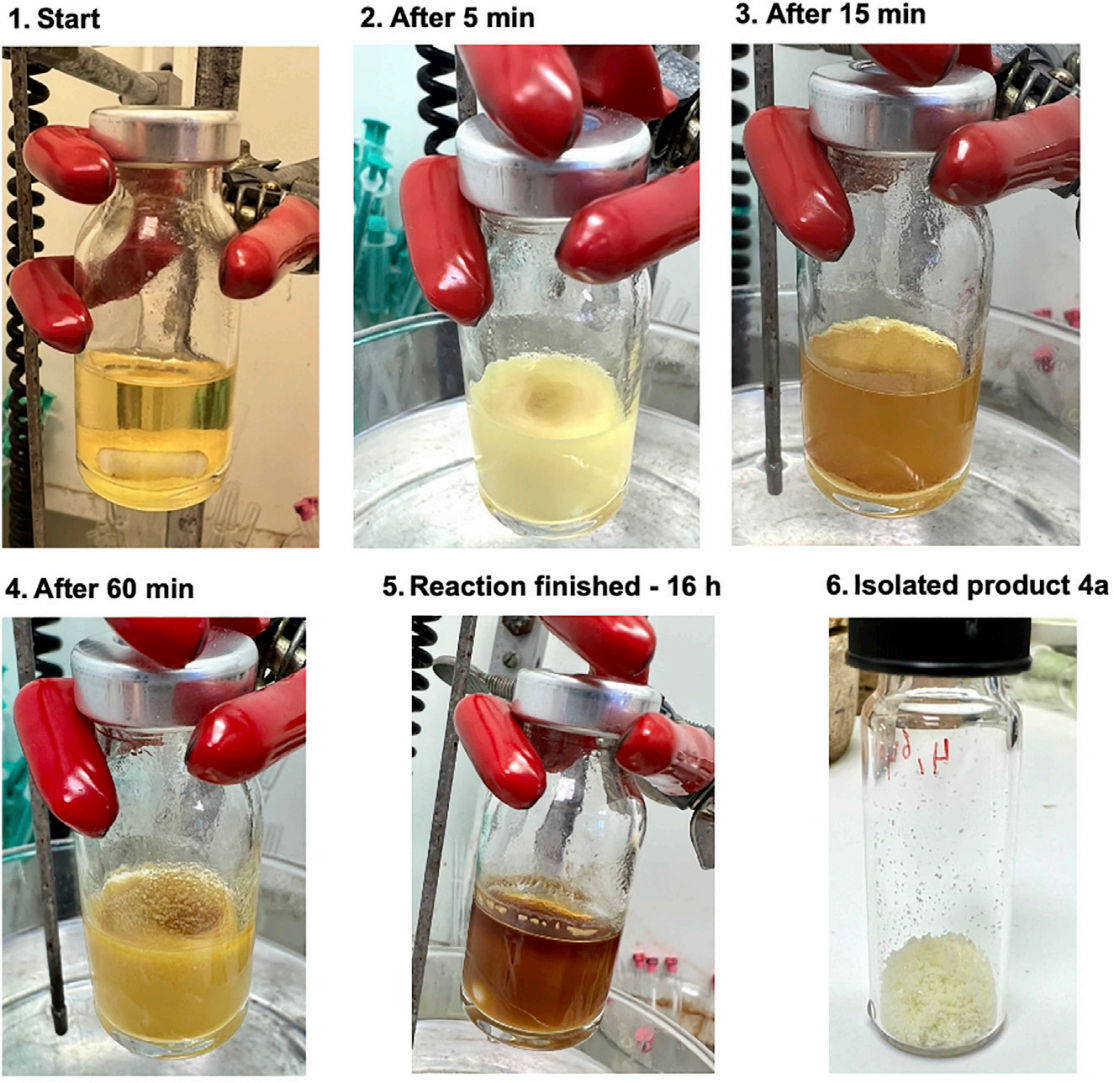
Reaction and appearance of product **4a** at different stages of the reaction

Step 5g: The water diarylation reaction stops after the first arylation step. This can be detected easily since this stage of the reaction results in a large amount of off-white precipitate in the reaction solvent, see stage 4 of Figure 2.

### Potential solution

Increase the temperature of the oil bath to 70°C and continue the stirring for 6 h. This will push the reaction to completion by enhancing the rate of the second intramolecular arylation. This will result in the reaction appearance to go from stage 4 of Figure 2 to the desired stage 5.

### Problem 4

Step 6i: The yield of the water diarylation is lower than expected.

### Potential solution

Degass the solvent and water again and ensure that the reaction is performed under inert atmosphere. The presence of oxygen in the reaction reduces the yields significantly and results in decomposition of the diaryliodonium salts, which can be seen in the crude in the form of the reduced ArI (4-fluoro-3-iodonitrobenzene). The reason for this is not fully understood.

## Resource availability

### Lead contact

Further information and requests for resources and reagents should be directed to and will be fulfilled by the lead contact, Berit Olofsson (Berit.Olofsson@su.se).

### Materials availability

This study did not generate new unique reagents, all compounds have been described in the original article; see Linde et al. (2022).

## Data Availability

The published article includes all [datasets/code] generated or analyzed during this study, see Linde et al. (2022).
